# I? AI? EXPLORING ARTIFICIAL INTELLIGENCE USAGE, PERSONALITY AND OTHER CHARACTERISTICS IN OLDER ADULTS

**DOI:** 10.1093/geroni/igae098.4213

**Published:** 2024-12-31

**Authors:** Sharon Ost-Mor, Nir Wittenberg, Yossefa Birati

**Affiliations:** University of Haifa, University of Haifa, Hefa, Israel; Ariel University, Ariel University, HaMerkaz, Israel; University of Haifa, Haifa, HaZafon, Israel

## Abstract

As Artificial intelligence (AI) becomes increasingly prevalent in modern society, understanding its adoption among older adults is crucial. Therefore, this study examines the relationship between AI usage, Big 5 personality traits, and positive solitude, among adults aged 60 and over in Israel. Grounded in the Technology Acceptance Model (TAM), the study explore how personality traits and positive solitude influences perceived AI usability and adoption. A sample of 260 older adults participated in the study. Data were collected through online validated questionnaires and open-ended questions, using Qualtrics. Results revealed that AI users (mean age 69.91, SD=6.7) were younger than non-users (M=74.91, SD=7.6). Of the AI users, 68% were women, 94% were home-dwelling, 65% married, and 98% independent. Additionally, 76% reported using AI for 30-120 minutes daily. Non-users felt subjectively older both physically (M=62.82, SD=11.7) and mentally (M=62.82, SD=10.7) compared to users (physical: M=54.84, SD=13.5; mental: M=58.65, SD=12.7). Significant correlations were found between attitude to life and perceived ability to use AI (r=0.315, p< 0.01), as well as between attitude to life and aging perception (r=0.495, p< 0.01). The qualitative analysis revealed varied responses to AI use. Participants noted significant benefits, including new opportunities and skill enhancement. However, they also encountered technical challenges, concerns about data reliability, and difficulties in formulating effective queries. These findings offer valuable insights into promoting AI adoption among older adults, potentially enhancing their quality of life and independence in the digital age. Future research should explore interventions to increase AI acceptance and usage among this population.

